# On the Rejected Vaccination Bill

**Published:** 1815-08

**Authors:** John Walker

**Affiliations:** Director of the Royal Jennerian and London Vaccine Institutions. Bond-Court, Walbrook


*For the London Medical and Physical Journal.*

*On the rejected Vaccination Bill;* by J. Walker, M.D.

FRIEND, wilt thou give me leave to congratulate a multitude of thy medical readers, who might have been allured, and some of whom I know would have been allured, into a situation which would have degraded them, on the escape which Lord Stanhope has just effected for them in the Upper, and on this occasion we may fairly say the wiser House of Parliament.

They would have been allured by the bait of the mammon of unrighteousness; for in England and Wales they might have litigiously obtained parochial remuneration, and, in Ireland, county charges. The National Vaccine Establishment lately attempted to obtain, from the legislature, the exclusive authority to grant licences for vaccinating, with the power of publishing the misdemeanours of their licensed inoculators in the country papers where they reside. It must excite surprize in all those who take time to think on this subject, that the censors of the College of Physicians, and the governors of the College of Surgeons, should be so lost to the recollection of the rights of Englishmen, as to attempt to obtain such arbitrary power over that class of them, who every where do often bear a part in the most secret and sacred concerns of domestic society—I mean the medical practitioners. The fact is, that this establishment embraces more than the censors and governors of annual appointment; and the permanent officers, usefully, perhaps, engaged in finding out and prosecuting the propagators of the deleterious disease, had actually managed to get the preposterous bill through the lower house, which was so instantly rejected in the House of Lords. The Bill (speciously entitled) "for ensuring the Benefits of Vaccination to such Poor Persons as are desirous thereof," made no provision for, no mention

---

even of the poor of North Britain. For those of Ireland, it seemed to make provision. They might apply to the surgeon of any county hospital, infirmary, or dispensary, (which *par parenthese* might be fifty long Irish miles from their residence,) and such surgeon (said the bill) shall, and is hereby required to vaccinate, &c.

The Commons had not, in their bill, assumed the arbitrary power of enacting any penalty for non-compliance with their orders; but they inadvertently had granted to the Board of the National Vaccine Establishment, the power to ruin their licensed inoculators, by advertising what they might please to term his misdemeanors in the paper read by all his patients.—Farewell.

*Bond-court, Walbrook;* JOHN WALKER, M.D.

13, *vii.* 1815. *Director of the Royal Jennerian and London Vaccine Institutions.*

